# Ten simple rules for training by researchers for researchers in a rapidly evolving workforce

**DOI:** 10.1371/journal.pcbi.1013408

**Published:** 2025-09-04

**Authors:** Meirian Lovelace-Tozer, John Brown, Robert Clemens, Kathryn Greenhill, Fathima Haseen, Danny Kingsley, Ellen A. Lyrtzis, Katherine Mills, Giorgia Mori, Kathryn M. Steel, Liz Stokes, Kathryn Unsworth, Adeline L. H. Wong, Amany Gouda-Vossos

**Affiliations:** 1 Australian Research Data Commons (ARDC), Melbourne, Victoria, Australia; 2 Curtin University, Perth, Western Australia, Australia; 3 Pawsey Supercomputing Research Centre, Perth, Western Australia, Australia; 4 CSIRO, Canberra, Australian Capital Territory, Australia; 5 The Australian National University (ANU), Canberra, Australian Capital Territory, Australia; 6 University of Western Australia, Perth, Western Australia, Australia; 7 Australian BioCommons, Melbourne, Victoria, Australia; 8 Federation University Australia, Churchill, Victoria, Australia; Carnegie Mellon University, UNITED STATES OF AMERICA

## Introduction

As we enter the fourth research paradigm, as evidenced by rapid advancements in the scientific methodology involving data-intensive practices [1–3], upskilling the next generation of researchers has become pivotal.

If you are a researcher, you will likely assist others in learning new skills, from computational tools to different data analysis methods. As experts in the field, researchers play an important role in upskilling the workforce. Upskilling often happens organically and informally through mentorship and collegiality, but a more structured approach would be vastly beneficial and impactful to those needing to upskill. Researchers are uniquely positioned to share their knowledge, experience and real-life challenges—this is key to successful training. When organizing a skills event for others, you may require guidance on where to begin.

Short-format training (SFT) provides an efficient medium to meet the demands of a fast-evolving workforce and allows researchers to stay informed and identify opportunities for rapid and ongoing skill enhancement. SFT refers to a non-formal workshop, short course, boot camp, or similar, that teaches skills and knowledge over a brief period, usually hours, days, or weeks [4]. Willams *et al*. called for SFT to be more reliable, effective, inclusive, and career-spanning in the face of rapid technological changes [5].

The following recommendations were inspired by the Australian Research Data Commons (ARDC) Digital Research Skills Summit 2023, that brought together Researchers, Learning Designers, Skills Trainers, and Librarians in productive discussions on how to run effective researcher skills training. The rules are intended to support researchers involved in upskilling their research community. Throughout this article, the term ‘trainer’ is shorthand for researchers designing and delivering training.

**Fig 1:**
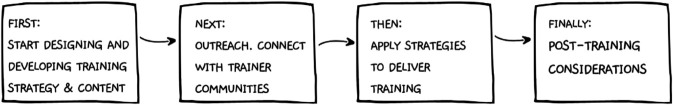
The basic steps of short-format training (SFT) development.

We have curated our ten simple rules into a streamlined workflow to assist in developing SFT (see Fig 1). Keep in mind that this process does not have to be linear. Developing successful training involves an iterative process, and consistent improvement based on feedback over time.

These rules outline how to think about skills learning for researchers, plan training sessions, and efficiently maximize learning. We offer recommendations on how to design and develop learner-centered training programs (Rules 1 and 2), foster outreach, and connect with trainer communities (Rules 3 and 4). We then provide tips to manage and optimize training (Rules 5, 6, and 7), and conclude with valuable insights on post-training considerations and continued learning (Rules 8, 9, and 10).

## Rule 1: Be learner-centered

A learner-centered training design considers the uniqueness of each learner and enables the trainer to play an impactful role in equipping individuals with the skills and knowledge necessary for success in their professional endeavors, ensuring equitable educational opportunities for all [6].

Learner diversity is a multifaceted and essential aspect of education and training. It encompasses a variety of backgrounds, cultural experiences, learning needs, abilities, and motivations. Recognizing and embracing this diversity is not just a moral imperative, it is also a fundamental teaching principle. It acknowledges that every learner brings unique strengths and challenges to the learning environment, making the educational experience richer and more dynamic. By valuing and accommodating this diversity, educators and trainers can create inclusive, authentic, and equitable learning spaces where all individuals have the opportunity to thrive and reach their full potential. This fosters an environment that celebrates the richness of human experiences and promotes learning outcomes that are both meaningful and impactful. The ultimate goal is to empower learners to become active participants in their learning process, and this is easier when learning experiences are authentic and relevant to the learner.

Implementing learner-centered SFT involves a structured approach that considers both the learner’s perspective and the course objectives [7–10]. The following eight steps summarize this structured approach.

**Fig 2:**
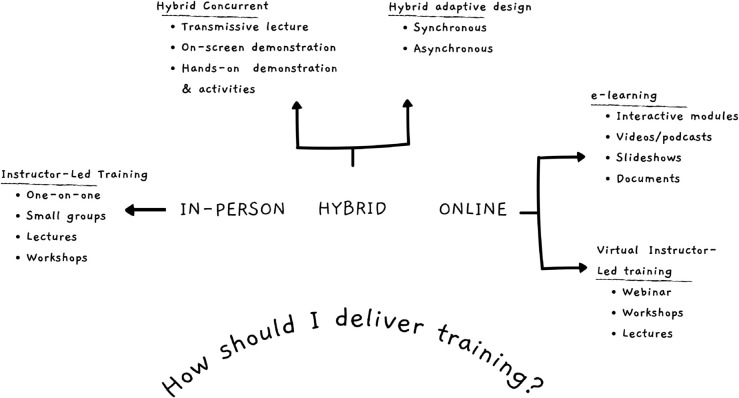
Modes for delivering short-format training (SFT).

1. **Define the overall goal and preliminary course description.** Start with the end in mind. What should learners be able to achieve by the end of the training?
2. **Define the audience** and characteristics of learners. Understand what appeals to them, their backgrounds, and their motivations.
3. **Define learning delivery modality.** With technology advancements, learners can choose their learning experience: in-person, online, hybrid, blended, self-paced e-learning, mobile learning, or virtual classrooms. See Fig 2 for various delivery modalities for SFT.
4. **Brainstorm ideas.** Collaborate with learners, subject matter experts, and learning specialists to understand what should be covered, how to do it, what will not be included, and to anticipate problems or misconceptions. Draw concept maps to visualize the course structure.
5. **Design and develop the course materials.** Use the results of step 4, and break down the content into manageable modules.
6. **Develop challenges** that allow learners to practice. Challenges serve as benchmarks for learners and trainers to gauge progress and identify areas needing further attention.
7. **Finalize the course description** to help the target audience understand if the course is suitable for them.
8. **Motivate learners** by organizing course content to align with their motivations. Demonstrating real-world relevance and applicability enhances learner engagement and success.

Using a learner-centered approach involves tailoring the experience to diverse learners’ individual needs and preferences, rather than following a one-size-fits-all approach. Keep the learners at the center of the learning process. A successful learning design prioritizes the individual needs and preferences of learners while aligning course content with their motivations and real-world applications. It is about fostering active participation, personalization, and inclusivity, ultimately resulting in more effective skills training.

## Rule 2: Use skills frameworks

A skills framework provides a structured outline of skills that individuals are expected to possess or develop in a particular context, such as in research. It categorizes various skills, often defining levels of proficiency.

Skills frameworks offer varied and structured methods to assist learners to develop an understanding of the specific skills they need, give perspective on their current skill level, and guide learners on when and how to effectively apply the skills and knowledge they acquire [6].

Skills frameworks provide trainers with an evidence-based process aligning learning goals with activities and structures to create motivating and stable environments for impactful learning. From an operational perspective, frameworks serve to ensure learners have the appropriate skills to succeed in their roles. They guide learners into roles where their existing skills can be leveraged, act as strategic tools to help learners and organizations pinpoint and address existing skills gaps, facilitate interconnections and linkages across diverse skills frameworks, and ensure the usage of a consistent terminology (taxonomy) underpinning a coordinated skills system [6].

From a learner perspective, frameworks create and identify structured training pathways and instill confidence that the skills they are acquiring are valuable and progress to future skills [6]. A framework is a way for learners to reach a shared understanding of what a skill, competency, or ability refers to and how particular skills relate to other skills.

Organizations with experience and a level of responsibility for providing training may create frameworks to ensure training occurs in a structured, progressive, and effective manner. Frameworks may cover a national or geographic area [11, 12]; be international and cover a broad range of occupational domains and disciplines [13]; focus on general fundamental employability skills [14]; or be for narrowly defined highly-skilled populations [15–17].

When developing SFT, you do not need to create a framework from scratch. Established frameworks provide a range of structured and tested approaches that can be used when developing training. For instance, the Skills Framework for the Information Age [13] and the Australian Core Skills Framework [14] include recommendations for applying frameworks:

- Assess and benchmark individual skills performance.
- Describe and identify skills and knowledge needs (skills gaps) relevant to the role, organization and sector.
- Create career-tailored approaches to training, teaching, and learning.
- Align curriculum to organizational/sector needs for improving employability.

The International Society for Computational Biology (ISCB) has developed a powerful tool to support trainers in implementing their framework [17]. Although designed for the field of computational biology, this is helpful across multiple domains and has been successfully used to support and inform various forms of training [18, 19].

**Fig 3:**
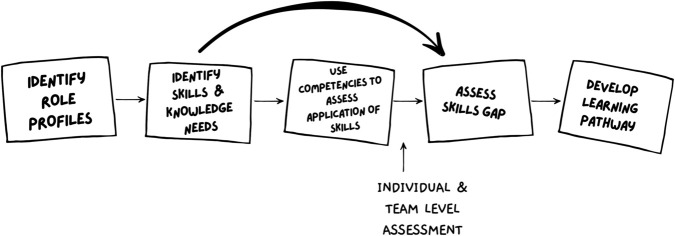
Skills framework development flow chart.

When applied within the workforce, the flow of training development starts through the identification of role profiles (or personas) to determine skills and knowledge needs, as well as competencies to assess the application of skills. From this, skills gaps can be determined and will inform the development of the learning pathway for SFT (see Fig 3).

## Rule 3: Harness existing resources and expertise

The key to efficiently upskilling researchers in a rapidly evolving workforce is to tap into available resources and expertise. Leverage the knowledge, tools, training materials, and talents already available to enhance your training capabilities and effectiveness. Researching existing resources forms part of training preparation and saves time and effort.

Furthermore, developing and leveraging relationships with external training organizations facilitates collaborations and partnerships focused on training requirements, enabling the delegation of specialized tasks tailored to expertise. Using external facilitators allows in-house expertise and resources to be redirected. Consultants or external organizations may deliver specialist skills that could not be provided in-house or to smaller cohorts. Universities and research organizations may also have sectoral training events available to their researchers and postgraduate students focusing specifically on the digital skills required by a cohort.

We highlight two case studies where existing resources and external relationships have been cultivated and leveraged to develop, share, and deliver SFT.

### 3.1. DReSA

Digital Research Skills Australasia (DReSA) is a skills and training registry that provides a free online space where researchers can connect with trainers in Australasia and discover digital research training events, resources, and providers to develop SFT [20].

DReSA is a gateway for researchers providing training to leverage relationships with experienced trainers online and to access and share training resources.

To ensure DReSA comprehensively covers skills offerings for an evolving workforce, trainers and training providers are invited to create an account, and register their resources. DReSA is also a platform for trainers to connect and collaborate on courses and materials to upskill the next generation of digital researchers.

### 3.2. ResBaz

The Research Bazaar (ResBaz) is a face-to-face, international festival promoting digital literacy at the center of modern research [21]. Since 2015, multiple-day festivals have been held annually at university campuses globally.

Researchers come together to upskill in ‘next-generation digital research tools and skills’. The event organization and training style accommodate researchers across diverse disciplines, fostering learning, camaraderie, and exchange of knowledge and skills. Collaborative face-to-face interactions create and nurture external relationships.

## Rule 4: Connect with trainer communities

Engaging with trainer communities is a great way to learn and understand the underpinnings of successful SFT, and to avoid training in isolation. A supportive and inclusive culture will enable you to align learning needs, common goals, and mindsets [22].

Strong outreach and community infrastructure are essential to supporting trainer uplift. They facilitate peer-to-peer supported learning and sharing of ideas and feedback, which can support trainer satisfaction and training longevity (Rule 8). Take advantage of the opportunity to shadow fellow trainers and invite others to observe your training. This collaboration promotes idea exchange and facilitates testing of new training approaches. In a rapidly evolving workforce, connecting with communities that focus on digital training delivery will ensure you stay up-to-date with training best practices.

The Carpentries and RLadies are examples of training communities focused on digital upskilling that can be engaged for advice on SFT development.

### 4.1. The Carpentries

The Carpentries’ purpose is to “teach foundational coding and data science skills to researchers worldwide" [23].

The Carpentries aim to spread data literacy and programmatic skills locally and globally by actively upskilling the instructional and technical skills of the instructor community. They foster an active and inclusive community by collaboratively developing openly-available lessons and delivering SFT using evidence-based teaching practices.

### 4.2. RLadies

RLadies is a global organization promoting gender diversity and inclusivity within the R programming community [24].

The organization provides educational resources, networking opportunities, mentorship, and training sessions, empowering members to excel in their careers and contribute meaningfully to the R community.

They embrace a collaborative approach to learning and knowledge sharing. Members share resources, tips, and best practices, fostering a culture of mutual support and growth. RLadies provides opportunities for organizers and members to enhance their teaching skills, expand their technical knowledge, and build their professional networks.

## Rule 5: Systematically manage logistics

When designing and delivering SFT the focus is often on content, while the management of administrative systems and logistics can be overlooked. Regardless of the size of the training, utilizing scheduling and registration systems can reduce the administrative burden pre- and post-training (especially if systems are integrated), allowing the trainer to focus on the learners and content.

Scheduling systems catalog all scheduled training activities at an organization, and can be used to promote upcoming SFT, while registration systems capture information about participants enrolling in scheduled training events.

For consistency and efficiency, it is important that scheduling and registration systems are interoperable. Platforms that integrate scheduling and registration include Learning Management Systems (LMS) such as Canvas and Moodle, video conferencing tools such as Microsoft Teams and Zoom, and event management systems such as Eventbrite and Whova. Check with your organization to see what scheduling and registrations systems are used and seek local support to help manage the process.

Systematic methods of cataloging delivered training allow trend identification, evaluation of strategy effectiveness, and the direction of resources based on demand. This can also inform future modes of learning (see Rule 1) and assist with the preparation to scale training (see Rule 9) [25, 26].

To effectively manage SFT and determine how it will be delivered (see Fig 2), it is helpful to consider:

- **Systems compatibility and integration:** Verify the platforms are compatible with your organization’s systems. Determine whether enterprise versions of the platforms are available, and if integration of systems is available. For example, formatting your training resource for future sharing on DReSA [20] will make it more discoverable without the administration hassle often associated with the process.
- **Requisite skills and knowledge:** Recognize the background skills and knowledge learners require to participate in the training. This also includes software needed before training commences or whether learners need assistance to install software. Consider demonstrating or providing resources to enable participants to navigate the platform and tools used consistent with learner-centered training (see Rule 1).
- **Communicating with registrants:** Decide how to update learners on training details. Some registration systems allow emails to be sent to registrants.
- **Accessibility:** Consider whether the systems are universally accessible, and what system functionalities can support learners with additional needs.
- **Training evaluation:** Determine how learners provide training feedback and how easy it is to extract training evaluation data in the preferred format (see Rule 7).

For an example list of the type of data collected in scheduling and registration systems, please see S1 Fig.

## Rule 6: Engage learners

As a training event approaches, the purpose of communication with learners switches from encouraging attendance to informing and preparing them for the learning experience. Maintaining interest and excitement by sending reminders, pre-work, expectations and sneak peeks (using the information gathered from the scheduling and registration platforms mentioned in Rule 5) increases retention [27, 28].

Once training commences, communication with learners is crucial to retain interest in training and to ensure maximum value and participation. This can be managed using the systems highlighted in Rule 5. Here are some examples trainers may enlist to reach learners: daily summaries for multi-day training, links to recordings and resources, activities, challenges, opportunities for questions, consultation, and networking. Create an inclusive environment where learners feel comfortable to ask questions, uphold a code of conduct, and avoid using dismissive language.

An impactful trainer keeps learners engaged through several teaching techniques within the classroom. Landmark work by Knowles, Holton, and Swanson in 2014 developed a framework that focuses on the individual learner differences and growth, outlining three dimensions of andragogy in practice [29]. Six core adult learning principles consider aspects and traits of the learners (learner needs, prior experiences, self-concept, readiness to learn, orientations, and motivations). This framework provides a systematic approach to the adult learning process and trainers can tailor these to SFT.

For an accessible approach, especially for new trainers, the Carpentries Instructor Training Curriculum [30] teaches the following strategies for instructors to employ in workshops:

- Trainer as motivator: Share the benefits of what learners will learn, and confidence in their ability to employ these skills.
- Align learning objectives that resonate with audiences; for instance using ‘authentic tasks’, which are real-world activities/problems they may encounter in their everyday lives or professional contexts.
- Prepare formative assessment to track learner progress.
- Seek feedback from learners and act on it throughout the workshop.
- Avoid demotivating learners by using dismissive language.

Allocate time for participants to interact and network with their colleagues by incorporating purposeful introductions at the start of training. This can promote collaboration, encourage idea exchange, and establish valuable connections.

Training is not complete once the session has been delivered. Learners may have questions about content from the training and may need a few weeks to process what they learned. From the trainer’s perspective, following up with learners can help obtain valuable feedback about the training and delivery for future improvements [27] (see Rule 7). Providing feedback can be confronting for some people so assure them it is confidential and anonymous.

## Rule 7: Improve through assessment and evaluation

Assessment is the measure of a learner’s skills and knowledge [31], while evaluation is the systematic process of collecting information and using it to improve future training [32]. Both are important indicators of learner performance and program efficacy, as they provide measurable observation.

What is delivered may not be received in the manner intended by trainers. It is important to prompt learners to reflect on their learning. Obtaining feedback from learners through formative assessment on the day helps to create impactful SFT that resonates. Consequently, learners may find SFT more relevant as it aligns with their needs and experiences. Planning the type of assessment and, subsequently, evaluation strategies to embed in training facilitates this process.

In SFT, self-assessment questions are frequently used as formative assessment: pre-training surveys, live quizzes, multiple-choice questions, and check-out questions at the end of a session. Trainers should also consider implementing challenges (see step 6 in Rule 1). Using assessment tools flexibly throughout training helps gauge learners’ comprehension. Examples include Curtin University’s ‘Research data management good practice self-assessment tool’ [33] and The Carpentries pre- and post-training surveys [34].

**Fig 4:**
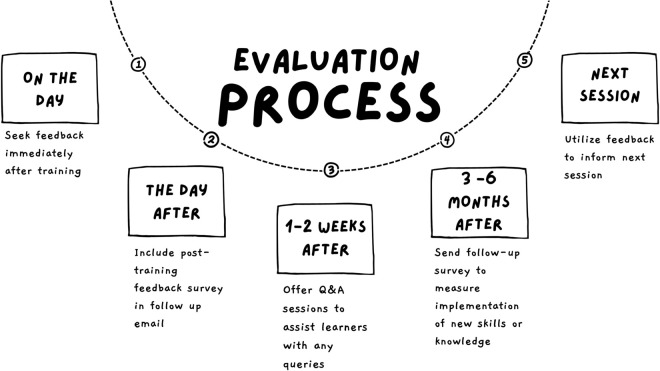
Evaluation process and timeline.

Complementary formative evaluation reviews training content and user experience and includes post-training feedback surveys and focus groups. Evaluating training provides an understanding of training delivery quality, and guides improvements in the learning experience [27, 32]. According to Kirkpatrick and Kirkpatrick [35], evaluation is best executed within a structured time range: immediately after each training session, one to two weeks after training, and three to six months after training (see Fig 4).

Training evaluation is designed to address various aspects of SFT, such as the impact on learners, learning outcomes, and training efficacy. Consider these questions when evaluating the impact of SFT on learners:

- Did the training achieve the intended outcomes?
- What needs to be changed for this training to be more effective?
- Is the content current?
- Were the delivery models/methods/platforms used appropriately?
- Does training frequency meet learner needs?
- Was the training pace suitable for learners?

Assessment and evaluation of training will:

- help determine whether the training achieved its objectives,
- make training efficacy visible,
- identify learning gaps,
- assist with planning for other future SFT, and
- provide an opportunity to collect learning metrics that reflect trainer value.

When reviewing the feedback, take the opportunity to self-reflect, and be mindful not to take some of the more negative comments to heart. Improve on things that are within your control to develop as a trainer.

## Rule 8: Consider trainer satisfaction and longevity

Attendees at the ARDC Digital Research Skills Summit 2023 highlighted the necessity of systemic changes in the research workforce to professionalize instructor training and mitigate trainer turnover. Systemic changes require the identification, creation, and explicit recognition of training roles within the research community. This also entails normalizing the acknowledgment of training providers in research outputs, akin to acknowledging funding sources, editorial support, and other types of contributions.

The unpredictability of trainer turnover can lead to efforts being redirected towards the development and upskilling of new trainers. Turnover poses risks to the capacity to deliver quality SFT.

Increasing and maintaining trainer satisfaction can mitigate turnover. However, even well-designed and planned SFT encounters trainer turnover, so succession planning is vital to ensure program sustainability [36, 37]. Attendees’ insights at the ARDC Digital Research Skills Summit 2023, resulted in the following strategies.

### Strategies for increasing trainer satisfaction

- Utilize professional training opportunities and determine whether training delivery activities count toward career progression.
- Recognize and foster the importance of the trainer’s role.
- Invite contributions to the development of training materials and recognize contributors.

### Support for training longevity

- Call for volunteer trainers and provide appropriate reward and recognition.
- Offer internships and mentoring.
- Build local or collaborative networks to coordinate specific types of training.
- Upskill existing trainers and pay them appropriately for the training they develop and deliver.
- Locate and use open source training materials to reduce the time and effort involved in developing training programs and refocus it towards delivery of the training.
- ‘Train the trainer’ sessions can benefit both existing and new trainers [30, 38, 39].

Many of these suggestions can be achieved by connecting with trainer communities (Rule 4).

## Rule 9: Be FAIR to enable scale

As training demands increase, relying on already developed materials will be essential not just for others, but also for your future SFT. This process can be facilitated by trainers making materials findable, accessible, interoperable, and reusable (FAIR) [40, 41].

Documentation, including the materials used for training and accompanying notes, is an excellent starting point to create resources for a larger audience, repeated deliveries, or self-directed learning [42]. When identifying aspects that may be reused in later deliveries it is worth considering the format and methodology of training (Rule 2), the technology used (Rule 5), and even the layout of the room for an in-person event (Fig 2).

Ownership and intellectual property in training can affect the approach to scaling. It may be sufficient to simply allow trainers and learners to share and use training materials. It is good practice to make materials findable and accessible by submitting them to an open access repository and applying an open reuse license for clarity of use and ease of citation. For more information, see the FAIR guidelines [41], and Garcia *et al*.’s guide for making training materials FAIR [40]. For consistency, it may be necessary to maintain oversight of who is delivering the material and to support their progress with scaling their delivery.

Designers of similar training can benefit from openly sharing training resources by working collaboratively to strengthen original material together, or pool efforts to produce a single new scaled-up training package combining their material. Providing opportunities for trainers to share experiences and to ask one another questions supports this. DReSA [20] is an example of an effective channel for encouraging and supporting collaborative efforts.

## Rule 10: Recognize training spans careers

Upskilling is not complete once SFT has been delivered, and this is particularly true within the workforce. Career-spanning training is imperative to keep researchers in line with current trends, and new techniques. The responsibility for professional development and learning ultimately falls to the individual, their self-efficacy [43] and mindset [44].

At the ARDC Digital Research Skills Summit 2023, attendees identified the importance of recognizing a ‘vision for future success’ when upskilling researchers, as this will ultimately lead to supportive learning cultures and continuous uptake of necessary training. Researchers as Skills Trainers play a critical role in supporting career-spanning training to meet the skills needs of a rapidly evolving workforce.

When developing SFT, take a moment to recognize the greater impact you create. As researchers, there are many measures you can use to assess the impact you have within your discipline (for example: publications and grants). Although harder to measure, training the next generation of researchers should not be understated, and will have deep ripple effects within your discipline and the broader research workforce.

Williams *et al*. have reiterated the importance of career-spanning training and identified a list of actions to strengthen its impact [5]. Throughout this article, we have provided tips to guide the development of SFT, which is one of the most effective methods of delivery due to its low cost, time commitment, and flexibility to adapt to unexpected changes. For a holistic approach to upskilling the research workforce, alongside SFT, encourage learners to engage in additional forms of career-spanning training (see S2 Fig).

In essence, the integration of learning throughout careers enables the collective workforce to rapidly adapt to cutting-edge technological changes and gives researchers opportunities to evolve their skills.

## Conclusion

Based on constructive discussions with participants at the ARDC Digital Research Skills Summit 2023, SFT must be accessible, scalable, and sustainable. Although these rules are broadly applicable to Skills Trainers, this article specifically targets researchers involved in training. Researchers play a crucial role in educating and mentoring their peers, offering training in research methodologies, data analysis, data management, dissemination of findings, and broader digital research practices.

By incorporating the rules outlined in this article, researchers can design and deliver effective skills training programs that continually enhance the capabilities of the greater research community. Through engaging with these guidelines, researchers providing training can collectively build a culture of lifelong learning and skill development within the research community.

## Supporting information

S1 Fig Planning checklist. Checklists for planning SFT, listing data to include in the scheduling system and registration system. Clip art used under license from Canva.
(PNG)

S2 Fig Types of career-spanning training. Short descriptions of other types of training besides SFT. Clip art used under license from Canva.
(PNG)
