# Supplementary figures and images for: Ten simple rules for training by researchers for researchers in a rapidly evolving workforce
Source: PLoS Comput Biol. 2025 Sep 4;21(9):e1013408. doi: 10.1371/journal.pcbi.1013408 (PMC12410803; doi:10.1371/journal.pcbi.1013408)

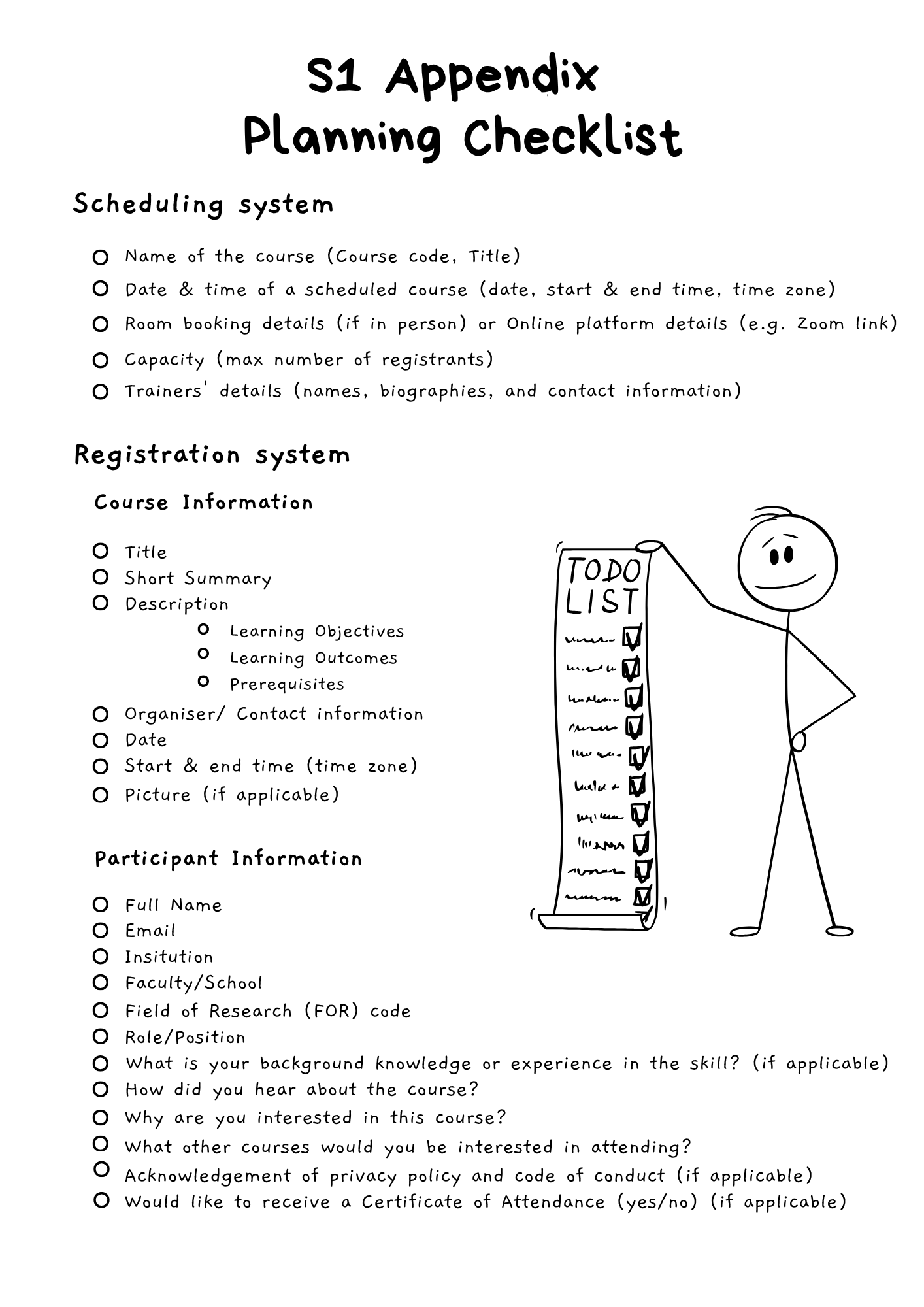

Supplement: S1 Fig — Checklists for planning SFT, listing data to include in the scheduling system and registration system. Clip art used under license from Canva. (PNG) [file pcbi.1013408.s001.png]

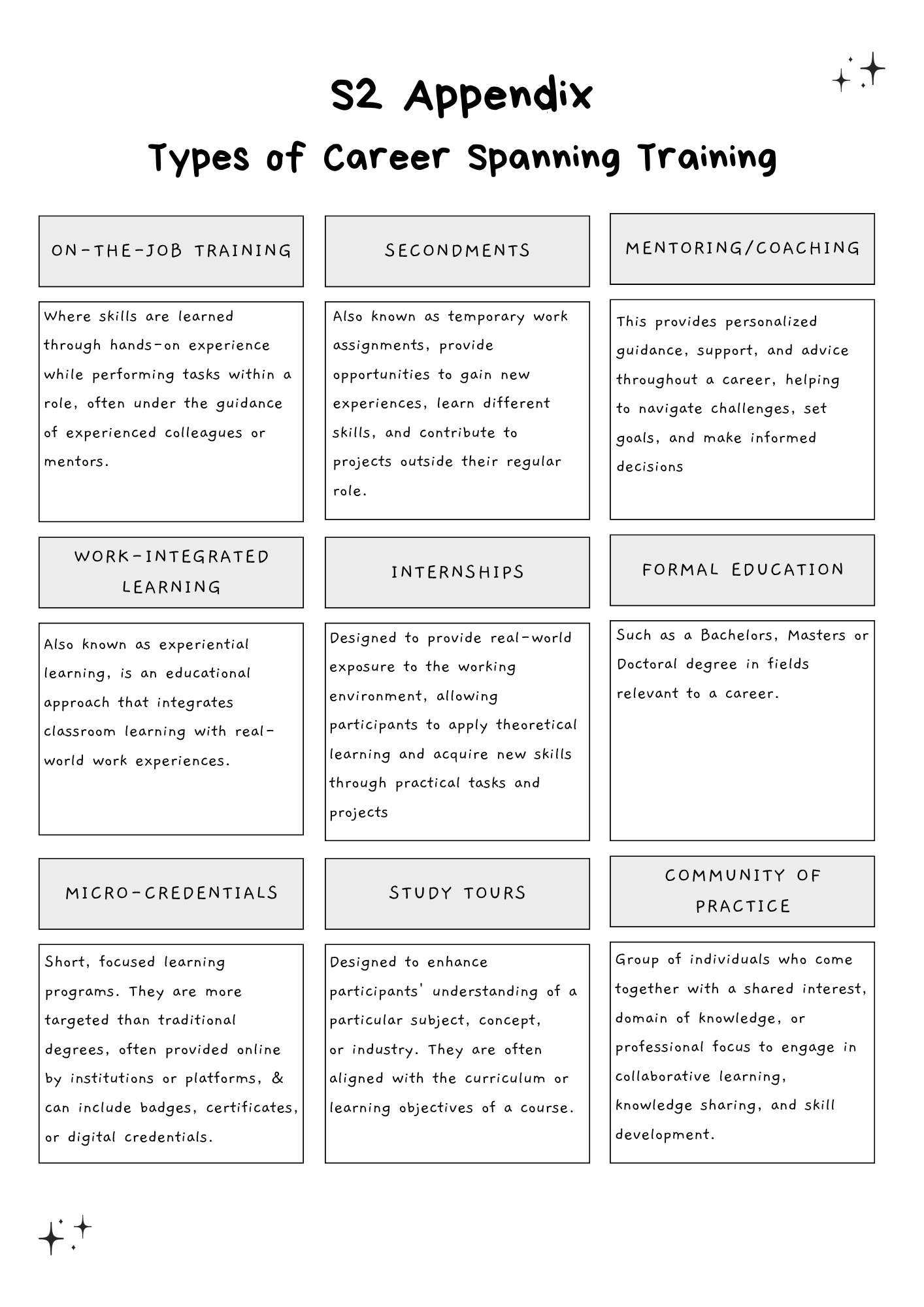

Supplement: S2 Fig — Short descriptions of other types of training besides SFT. Clip art used under license from Canva. (PNG) [file pcbi.1013408.s002.png]
